# Heritable Variation of Foliar Spectral Reflectance Enhances Genomic Prediction of Hydrogen Cyanide in a Genetically Structured Population of *Eucalyptus*

**DOI:** 10.3389/fpls.2022.871943

**Published:** 2022-03-31

**Authors:** Paulina Ballesta, Sunny Ahmar, Gustavo A. Lobos, Daniel Mieres-Castro, Felipe Jiménez-Aspee, Freddy Mora-Poblete

**Affiliations:** ^1^The National Fund for Scientific and Technological Development, Talca, Chile; ^2^Plant Breeding and Phenomic Center, Faculty of Agricultural Sciences, Universidad de Talca, Talca, Chile; ^3^Department of Food Biofunctionality, Institute of Nutritional Sciences, University of Hohenheim, Stuttgart, Germany; ^4^Institute of Biological Sciences, University of Talca, Talca, Chile

**Keywords:** genomic and phenomic prediction, genomic heritability, defense chemistry, spectral reflectance indexes, spectroradiometer, specialized metabolite

## Abstract

Plants produce a wide diversity of specialized metabolites, which fulfill a wide range of biological functions, helping plants to interact with biotic and abiotic factors. In this study, an integrated approach based on high-throughput plant phenotyping, genome-wide haplotypes, and pedigree information was performed to examine the extent of heritable variation of foliar spectral reflectance and to predict the leaf hydrogen cyanide content in a genetically structured population of a cyanogenic eucalyptus (*Eucalyptus cladocalyx* F. Muell). In addition, the heritable variation (based on pedigree and genomic data) of more of 100 common spectral reflectance indices was examined. The first profile of heritable variation along the spectral reflectance curve indicated the highest estimate of genomic heritability ($h_{g}^{2}$=0.41) within the visible region of the spectrum, suggesting that several physiological and biological responses of trees to environmental stimuli (ex., light) are under moderate genetic control. The spectral reflectance index with the highest genomic-based heritability was leaf rust disease severity index 1 ($h_{g}^{2}$=0.58), followed by the anthocyanin reflectance index and the Browning reflectance index ($h_{g}^{2}$=0.54). Among the Bayesian prediction models based on spectral reflectance data, Bayes B had a better goodness of fit than the Bayes-C and Bayesian ridge regression models (in terms of the deviance information criterion). All models that included spectral reflectance data outperformed conventional genomic prediction models in their predictive ability and goodness-of-fit measures. Finally, we confirmed the proposed hypothesis that high-throughput phenotyping indirectly capture endophenotypic variants related to specialized metabolites (defense chemistry), and therefore, generally more accurate predictions can be made integrating phenomics and genomics.

## Introduction

Plants produce an outstandingly wide diversity of specialized metabolites (or secondary metabolites), fulfilling a wide range of biological functions, and helping plants to better cope with abiotic and biotic factors. These molecules are usually divided into three groups, including phenolic compounds, terpenes, and nitrogen-containing compounds (Zeng et al., 2020; Balestrini et al., 2021; Mieres-Castro et al., 2021). The major classes of nitrogen-containing specialized metabolites in the plant kingdom are cyanogenic glucosides, alkaloids, and non-protein amino acids. In particular, cyanogenic glycosides are present in various trees and plants, many of which are utilized as a source of food for humans and animals (Gleadow and Møller, 2014). These specialized metabolites are part of a plant’s strategy against herbivores and therefore play a major role in the ecosystem as defense-related compounds (Zenk and Juenger, 2007). The endogenous plant enzymes can react with cyanogenic glycosides and release hydrogen cyanide (hereafter referred to as HCN), a process known as cyanogenesis, which may be toxic to generalist herbivores and pathogens (Appenteng et al., 2021). Therefore, the effectiveness of cyanogenesis is a phytochemical defense strategy, dependent on cyanogenic plants’ capability to release HCN in sufficient quantities to be considered toxic.

There are over 3,000 cyanogenic plant species, representing more than 130 families, including Fabaceae, Leguminosae, Myrtaceae, Rosaceae, and many others (Gleadow and Møller, 2014; Appenteng et al., 2021; Mora-Poblete et al., 2021). In addition to serving as important compounds, cyanogenic glycosides play multiple roles in physiological functions involved in phenotypic plasticity during specific developmental stages and particularly under challenging environmental conditions, such as drought (Gleadow and Møller, 2014; Pičmanová et al., 2015; Rosati et al., 2019). In fact, cyanogenic glycosides can play an important role in primary metabolism processes as nitrogen and glucose transporters (Møller, 2010). The metabolic profile in cyanogenic species is modulated according to the interaction between genes and environmental conditions, resulting in a physiological trade-off between the production of defense metabolites and growth-related tasks (Gleadow and Woodrow, 2000; Gleadow and Møller, 2014).

The biosynthesis, degradation, biological functions, polymorphism, and regulation of cyanogenic glycosides have been widely studied in plant species (Sun et al., 2018), such as sorghum (Darbani et al., 2016), cassava (Zidenga et al., 2017), *Prunus* spp. (Thodberg et al., 2018), and *Eucalyptus* spp. (Goodger et al., 2004; Hansen et al., 2018). More than 20 species of the *Eucalyptus* genus have been identified to be cyanogenic (Gleadow et al., 2008), which serves as a powerful experimental system to study cyanogenesis as a chemical defense response against herbivores and pathogens. Among the *Eucalyptus* species studied to examine their cyanogenic content are *E. nobilis*, *E. polyanthemos*, *E. yarraensis*, *E. dalrympleana*, *E. camphora*, *E. viminalis*, and *E. cladocalyx* (Gleadow et al., 2003, 2008; Goodger et al., 2004; Neilson et al., 2006; Hansen et al., 2018). Gleadow et al. (2003) and Neilson et al. (2006) reported concentrations of HCN ranging from 0 to 0.153 mg HCN g^−1^ dw and from 0.01 to 0.5 mg HCN g^−1^ dw in leaves of *E. nobilis* and *E. camphora*, respectively. In *E. polyanthemus*, Goodger et al. (2004) found that these compounds can vary from 0.002 to 0.2 mg HCN g^−1^ dw in leaves by supplementing the plants with different concentrations of nitrogen. Moreover, prunasin concentration is highly variable depending on tissue type and leaf age in *E. cladocalyx* (Gleadow and Møller, 2014). In fact, prunasin concentration of less than 20 ug HCN g^−1^ dw can be found in adult leaves, while a concentration of 60 ug HCN g^−1^ dw can be found in immature flower buds (Hansen et al., 2018). Furthermore, Mora-Poblete et al. (2021) reported that the concentration in adult leaves can reach up to 1.5 mg HCN g^−1^ dw under dry land conditions. Different types of cyanogenic glycosides have been identified in *Eucalyptus* trees, such as prunasin (the predominant type); amygdalin; sambunigrin (the epimer of prunasin); neoamygdalin (the epimer of amygdalin); and eucalyptosin A, B, and C (Gleadow et al., 2008; Neilson et al., 2011; Hansen et al., 2018). These specialized metabolites are synthesized from phenylalanine, a process in which several cytochrome P450s (CYP) proteins are involved, to produce monoglycosides (Yamaguchi et al., 2014; Hansen et al., 2018). Particularly, defense mechanisms against pathogens and pests have rarely been reported in cyanogenic *Eucalyptus* spp., unlike other crops (Yactayo-Chang et al., 2020). On the other hand, it has been shown that the biosynthesis of these metabolites is induced by different abiotic factors (i.e., soil nutrients and water level, light, and temperature; Gleadow and Woodrow, 2002; Goodger et al., 2002, 2004; Simon et al., 2010).

In *E. cladocalyx*, prunasins were mainly found in floral and vegetative tissues, along with amygdalin in a minor concentration (Hansen et al., 2018). In this cyanogenic species, three CYP proteins and one UDP-glucuronosyltransferase protein (UGT) are involved in prunasin biosynthesis. First, L-phenylalanine is converted into phenyl acetaldoxime by CYP79A125 and is then dehydrated into phenyl acetonitrile by CYP706C55. Phenyl acetonitrile is converted into mandelonitrile by CYP71B103. Subsequently, UGT85A59 catalyzes the conversion of mandelonitrile into prunasin. In other cyanogenic plants, it has also been reported that another UGT protein catalyzes the conversion of cyanogenic monoglycosides (such as prunasin) into diglycosides (amygdalin; Yamaguchi et al., 2014; Del Cueto et al., 2017; Thodberg et al., 2018).

Despite the importance of cyanogenic glycosides as a source of defense-related compounds, and the advances made in the biosynthesis, catabolism, transport, and storage of these specialized metabolites, few published works have focused on the prediction of the amounts of cyanogenic glycosides in *Eucalyptus* trees. The complex nature of the genetic architecture of cyanogenic glycoside content may be a bottleneck for genomic prediction studies, especially when high-density marker panels are not available or when study species are poorly represented in commercial DNA arrays (Aguirre et al., 2019; Ballesta et al., 2020; Lebedev et al., 2020). According to Pryce et al. (2014), the use of low-density marker panels will inevitably affect the accuracy of the genomic prediction of target traits to some degree. In this sense, Mora-Poblete et al. (2021) found a moderate predictive ability for HCN content (of up to 0.47) in a genetically structured breeding population of cyanogenic *E. cladocalyx*, using a low-density marker panel of single nucleotide polymorphisms (SNPs) and haplotype blocks constructed from them, which had a better predictive ability (slightly higher) than the individual SNPs for predicting HCN content in leaves. Consistently, these findings are in line with other studies, which have concluded that the use of haplotypes, instead of individual SNPs, could have a higher predictive ability of certain quantitative traits (Contreras-Soto et al., 2017; Ballesta et al., 2019; Valenzuela et al., 2021). Consequently, in the present study, we examined a strategy of prediction that integrates conventional pedigree information, genomic data, and high-throughput phenotyping techniques, which is an effective method with which to break through the bottleneck of low-density marker panels.

Similarly, an integrated genomic and phenomic selection strategy that has been performed in breeding programs of plants and forest trees (Rincent et al., 2018; Krause et al., 2019), in which spectral reflectance data are used as regressors (or reflectance-derived relationship matrices) for accelerating the breeding progress of complex traits. Interestingly, Rincent et al. (2018) and Krause et al. (2019) observed that prediction models using spectral signatures performed similarly or superior to marker- and pedigree-based genomic selection models when predicting within and across environments. We hypothesized that high-throughput phenotyping platforms could indirectly capture endophenotypic variants, which could be related to specialized metabolites, and, therefore, we expected to be able to perform a more robust prediction, considering the spectral reflectance relationship among the trees. Consequently, indirect endophenotypic measures could enable these phenomic prediction methods to be incorporated into studies of specialized metabolites, such as cyanogenic glycosides. Additionally, we presented the first profile of heritable variation (genomic- and pedigree-based approaches) in the leaves of cyanogenic *Eucalyptus* (*E. cladocalyx*) along the spectral reflectance curve for each 1-nm wavelength interval (from 400 to 2,400 nm). The genomic- and pedigree-based heritability of the most widely used spectral reflectance indices (SRIs) was also estimated, such as the photochemical reflectance index (PRI), the green normalized difference vegetation index (GNDVI), water-related SRIs, the normalized difference vegetation index (NDVI), and the normalized pigment chlorophyll ratio index (NPCI), among others. This represents the first exploration of the genomic variation along the reflectance curve in a tree species and supports high-throughput phenotyping as a suitable approach for the prediction of specialized metabolites.

## Materials and Methods

### Plant Materials

The study was carried out in a provenance/progeny trial of *Eucalyptus cladocalyx*, established in 2001, and situated in the southern Atacama Desert, in Chile, Choapa Province (31° 55′ S, 71° 27′ W, 167 m.a.s.l.). The climate in this area is classified as predominantly arid, according to the De Martonne aridity index (Arriagada et al., 2018). The trial consisted of a genetically structured population with 49 half-sib families (details in Valenzuela et al., 2021) according to a randomized complete block design, with 30 blocks and single-tree plots.

The concentration of hydrogen cyanide (HCN) was determined in the leaves of 310 trees with three replications using the protocol developed by (Brinker and Seigler, 1989). Fully expanded and fresh mature leaves (~10 leaves per tree) were collected in the first third and from the northern side of the tree canopy (Woodrow et al., 2002). The methods for obtaining and quantifying HCN for each leaf sample can be reviewed with details in Mora-Poblete et al. (2021). Briefly, a hydrolysis of cyanogenic glucosides from plant tissue was performed, trapping the resulting HCN in a well containing 1 M NaOH. The cyanide captured was quantified using the König reaction. The final absorbance was measured at 595 nm using a Genesys 10UV spectrophotometer (ThermoSpectronic). The amount of cyanide was determined by interpolation into a calibration curve built with sodium cyanide (0.2–1.2 μg/ml, *R*^2^ = 0.9935). All samples were analyzed in triplicate, and results were presented as mean mg HCN equivalents g^−1^ dry weight (dw). The cyanide concentration was expressed as mg HCN equivalents g^−1^ dry weight (dw) primarily for the glucoside prunasin (Gleadow and Woodrow, 2000). The trees exhibited a HCN content from <0.0001 mg HCN g^−1^ dw up to 1.54 mg HCN g^−1^ dw in their leaves.

### Spectral Reflectance Assessments

Absolute reflectance measurements of leaves (0.1 g lyophilized leaf powder per sample) were performed using a portable FieldSpec^®^ 4 HiRes spectroradiometer (ASD Inc., Boulder, CO, United States), which covers the 350–2,500 nm range (the full range of the solar irradiance spectrum) with a 2.3-mm-diameter optical fiber. The spectral range between 350–399 and 2,400–2,500 nm was removed. RS3 software (ASD Inc., Boulder, CO, United States) was used to calibrate and control the spectrometer and acquire spectral signatures. The equipment was configured to integrate three samples per scan. The reflectance data were extracted using View Spec Pro 2008 software (ASD Inc., Boulder, CO, United States). The spectral data were pre-processed according to method of Rincent et al. (2018), in the R package Prospectr (Stevens and Ramirez-Lopez, 2014), in which the reflectance measures were normalized (centered and scaled), and their first derivative was computed using a Savitzky–Golay filter, with a window size of 37 data points.

The raw spectral reflectance data were also used to calculate more than 125 previously characterized spectral reflectance indices (SRIs), using either normalized or simple ratios of reflectance measures in the hsdar package (Lehnert et al., 2018). These SRIs have been shown to correlate with different physiological and biochemical components in plants and to provide information about several physiological (and agronomic) traits (Lobos and Poblete-Echeverría, 2017). A stepwise regression was conducted to evaluate and determine an appropriate number of predictor variables (SRIs) for HCN content. The optimal model was selected based on Akaike’s information criterion (AIC). Multicollinearity among SRIs was examined using the variance inflation factor (VIF). The VIF value for each SRI was interpreted as follows (Liu et al., 2019; Qian et al., 2019): 0 < VIF < 10, 10 ≤ VIF < 100 and VIF ≥ 100, indicating that there is no strong and severe multicollinearity, respectively.

### DNA Isolation and Genotyping

Genomic DNA was extracted from the leaves of individual samples of *E. cladocalyx*, according to the method of Ballesta et al. (2019, 2020)>. All individuals were genotyped using an array (Illumina Infinium) of ~60,000 single nucleotide polymorphisms (SNPs). SNP markers with a call rate < 90% and a minor frequency allele <0.05 were removed from the SNP data matrix. A total of 3,897 SNPs were retained, which were distributed across the 11 chromosomes (~ 350 SNPs per chromosome) of *Eucalyptus*, with a density of one SNP for every 11,000 bp. After applying these filters, haplotype blocks were constructed according to the solid spine method in Haploview v. 4.2 (Barrett et al., 2005), which were later used in the haplotype-based genomic prediction of HCN. Only haplotype blocks with a D ‘value ≥ 0.9 and a LOD score ≥ 2 were considered for the further analyses. According to previous studies, genomic prediction models based on SNPs forming haplotypes have a slightly higher predictive ability of HCN content than individual markers (Mora-Poblete et al., 2021). Therefore, this antecedent was considered in the present study, in such a way that all genomic prediction models were implemented using haplotypes as predictor variables, instead of SNP markers.

### Heritability in Single Wavelengths and Spectral Reflectance Indices of Leaf

The following prediction models were used to estimate the heritable variation along the reflectance curve and for all SRIs evaluated in *Eucalyptus* leaves:


(1) $$y=X\beta +Qv+Z_{1}g_{1}+\varepsilon$$

(2) y=Xβ+Pρ+Za+ε

where y corresponds to phenotype records, i.e., SRIs or spectral reflectance measurements for each wavelength. X, Q, Z, $Z_{1}$, and P are the incidence matrices of the associated vectors. β corresponds to the vector of the block effect (experimental design). v and ρ are the vectors of genetic population structure (Pritchard et al., 2000) and provenance (seed source) effects, respectively. $g_{1}$ is the vector of genomic values with $g_{1}$ ∼ *N*(0, *G_1_* $\sigma_{g_{1}}^{2}$), where *G_1_* is the genomic relationship matrix (based on filtered SNP data), and $\sigma_{g_{1}}^{2}$ corresponds to the genomic variance component. a is the vector of the polygenic background effects (based on pedigree information), in which a ∼ *N*(0, *A*$\sigma_{a}^{2}$), where *A* corresponds to the pedigree-based numerator relationship matrix and $\sigma_{a}^{2}$ is the additive genetic variance, and ε is the vector of residual effects, distributed as ε ∼ *N*(0, *I*$\sigma_{e}^{2}$), where *I* is the identity matrix and $\sigma_{e}^{2}$ is the residual variance. Heritability estimates (narrow sense heritability $h_{a}^{2}$ and genomic heritability $h_{g}^{2}$) of each wavelength measure and SRI were calculated according to the following formulas: $h_{a}^{2}=\frac{\sigma_{a}^{2}}{\sigma_{a}^{2}+\sigma_{e}^{2}}$ and $h_{g}^{2}=\frac{\sigma_{g_{1}}^{2}}{\sigma_{g_{1}}^{2}+\sigma_{e}^{2}}$.

### Prediction Models for HCN in *Eucalyptus* Leaves

The following prediction models were performed using the pedigree, genomic, and spectral information. The first model included only genomic data, as follows:


(3) $$y^{*}=Qv+Z_{2}g_{2}+\varepsilon$$

where $y^{*}$ corresponds to the vector of adjusted phenotypes (HCN) for block effects (experimental design). $Z_{2}$ is the incidence matrix associated with the $g_{2}$ vector, which corresponds to genomic values based on haplotypes (Mora-Poblete et al., 2021). The Model 4 can be expressed in matrix form as:


(4) $$y^{*}=P\rho +Za+\varepsilon$$

where the terms Pρ and Za are described in the 2.4 section (see Models 1 and 2). The Model 5 included the population genetic structure and polygenic effects (see above):


(5) $$y^{*}=Qv+Za+\varepsilon$$

The fourth prediction model (Model 6) combined the polygenic and genomic effects:


(6) $$y^{*}=Qv+Za+Z_{2}g_{2}+\varepsilon$$

The following model (Model 7) included the spectral data as regressors in the prediction model:


(7) $$y^{*}=Qv+Z_{3}h+\varepsilon$$

where, $Z_{3}$ is the incidence matrix associated to h vector, which corresponds to the vector of wavelength effects (Gonçalves et al., 2021). The prediction Model 8 comprised the genomic and wavelength effects:


(8) $$y^{*}=Qv+Z_{2}g_{2}+Z_{3}h+\varepsilon$$

Similarly, the Model 9 included the polygenic and wavelength effects:


(9) $$y^{*}=Qv+Za+Z_{3}h+\varepsilon$$

The Models 10, 11, and 12 included the SRIs effects, as follows:


(10) $$y^{*}=Qv+X_{i}\beta_{i}+Z_{2}g_{2}+\varepsilon$$

(11) $$y^{*}=Qv+X_{i}\beta_{i}+Z_{3}h+\varepsilon$$

(12) $$y^{*}=Qv+X_{i}\beta_{i}+Za+Z_{2}g_{2}+\varepsilon$$

where, $X_{i}$ is the incidence matrix for the $\beta_{i}$vector (fixed effect), which corresponds to SRIs’ effects (1, …, n). The $\beta_{i}$ vector contains a single SRI or multiple SRIs (selected according to the stepwise regression method); as a covariate (s) in the model. The Model 13 included the wavelength effects in combination with polygenic and genomic effects:


(13) $$y^{*}=Qv+Za+Z_{2}g_{2}+Z_{3}h+\varepsilon$$

The Model 14 corresponded to the full prediction model, which integrated all effects above described:


(14) $$y^{*}=Qv+X_{i}\beta_{i}+Za+Z_{2}g_{2}+Z_{3}h+\varepsilon$$

The following three Bayesian regression methods were used to predict the effect of each wavelength: Bayes B, Bayesian ridge regression (BRR), and Bayes C (Meuwissen et al., 2001; Gianola et al., 2006; Habier et al., 2011). These methods were selected because they have different analytical assumptions, which allows the analysis of traits with different genetic architectures, and are traditionally used in the context of genomic prediction. BRR method assumes that the regressors (For instance, SNPs or reflectance measurements) have a common variance and have the same contraction effect. The predictor effect ($m_{i}$) is distributed $m_{i}|\sigma_{m}^{2}\sim N\left(0,\sigma_{m}^{2}\right)$, and the common variance is distributed scaled- inverse Chi-squared ($\sigma_{m}^{2}|\alpha_{m},S_{m}\sim X^{-2}$($\alpha_{m},S_{m})$, where $\alpha_{m}$ and $S_{m}$ corresponds to degree freedom and scale parameters, respectively. Contrarily, Bayes B denotes that each regressor has its own variance, and uses a mixed distribution with a mass at zero, such that the prior distribution of the effects of the all regressors is assumed as:


$$m_{i}|\sigma_{mi}^{2},\pi =\left\{\begin{array}{c} 0\;\mathrm{with}\;\mathrm{probability}\;\pi \\ N\left(0,\sigma_{mi}^{2}\right)\;\mathrm{with}\;\mathrm{probability}\;1-\pi \end{array}\right.$$

The π parameter represents the probability that the regressor effect tends to be zero. Bayes C is a method that combines assumptions of Bayes B and BRR, in which the predictors effects have a common variance and it assigns a non-null prior probability for the predictor effect to be equal to zero (see more details about methods in Meuwissen et al., 2001, Gianola et al., 2006 and Habier et al., 2011). The Bayesian Generalized Linear Regression (BGLR) library in R (Pérez and De los Campos, 2014) was used for fitting all models and making predictions. The BGLR procedure considered a run with 1,000,000 iterations, a burn-in period of 100,000 and a thin of 50.

### Assessing Model Fitting and Predictive Ability

Prediction models 3 to 14 were evaluated and compared in terms of their predictive ability (PA) and goodness of fit using the deviance information criterion (DIC; Spiegelhalter et al., 2002). A DIC difference > 10 between two competitive models was considered to be supported against a model with higher DIC; a DIC difference between 3 and 10 was considered as substantial difference between models, while a difference < 3 was considered as not significant. All models were calibrated using ~300 individuals (which were also used for the estimation of genetic parameters) and validated considering the 20% of the total population. The PA of each model was calculated as the correlation between the adjusted phenotypes $\left(y^{*}\right)$ from the validation dataset and the predicted phenotypes (${\hat{y}}^{*}$; Ballesta et al., 2020; Mora-Poblete et al., 2021). A process of fivefold cross-validation was used to evaluate the PA of all models.

## Results

### Heritable Variation in Single Wavelengths and SRIs

**Figure 1:**
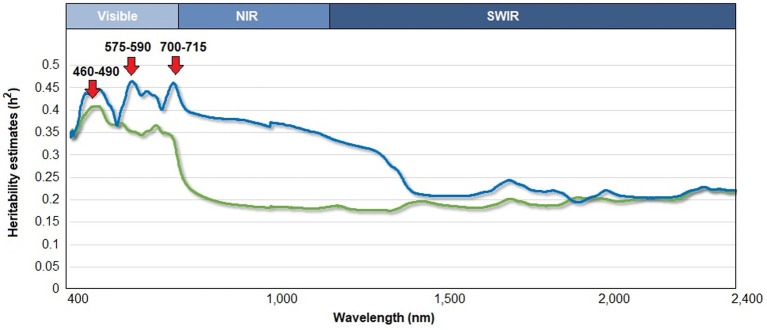
Estimation of pedigree-based heritability (blue) and genomic heritability (green) along the reflectance curve (in the range of 400–2,400 nm) in leaves of a cyanogenic species of *Eucalyptus*. The upper axis indicates the range of the three main regions of the electromagnetic spectrum: visible, near infrared (NIR), and shortwave infrared (SWIR).

The estimates of genomic heritability for 1-nm wavelength bands along the reflectance curve (400–2,400 nm) ranged from 0.18 to 0.41, whereas pedigree-based estimates varied from 0.19 to 0.46 (Figure 1). The pedigree- and genomic-based heritability patterns were highly correlated (Pearson’s coefficient, *r* = 0.60). The highest estimate of genomic heritability ($h_{g}^{2}$ = 0.41) was found within the visible region: 460–490 nm. The estimates of pedigree-based heritability peaked at two different wavelength regions. The first one was at 575–590 nm ($h_{a}^{2}$= 0.46; the visible region), and the second one was at 700–715 nm ($h_{a}^{2}$ = 0.46; the red edge spectral region).

The SRI with the highest genomic-based heritability was leaf rust disease severity index 1 (LRDSI1; $h_{g}^{2}$ = 0.58; Table 1; Supplementary Table S1), which was calculated with reflectance measurements at 605 and 455 nm, followed by the anthocyanin reflectance index (ARI) and the Browning reflectance index (BRI; $h_{g}^{2}$ = 0.54); whereas the least heritable index was the enhanced vegetation index (EVI; $h_{g}^{2}$ = 0.22), which was calculated using reflectance at 800, 670, and 475 nm. The index with the highest heritability based on pedigree was the REP_LE index ($h_{a}^{2}$ =0.48), which was calculated with reflectance measures within the red edge region, followed by the double difference Index (DD), REP_LE, the derivative index (D2), and the Datt index ($h_{a}^{2}$ = 0.47), whereas the least heritable index was the EVI index (EVI; $h_{a}^{2}$ = 0.18). According to the stepwise regression method, four spectral reflectance indices were significantly associated with HCN (*p* < 0.05); i.e., simple ratio 10 (SR10), normalized difference lignin index (NDLI), normalized difference nitrogen index (NDNI), and normalized pigment chlorophyll index (NPCI). The multiple regression adjusted by the three SRIs explained ~18% of the total variation (*p* = 7.336^*^10^−11^). The SR10, NDLI, NDNI, and NPCI indices explained ~10, 8, 2, and 6% of the total variation, respectively. In this way, these four SRIs were considered for predicting HCN. Heritability estimates based on pedigree and genomic data (SNP markers) were equivalent for the indices SR10 ($h_{a}^{2}$ = 0.41; $h_{g}^{2}$ = 0.41) and NDNI ($h_{a}^{2}$ = 0.28; $h_{g}^{2}$ = 0.29; Table 2). The pedigree-derived relationship matrix accounted for a greater portion of the NDLI index variation than the SNP-derived relationship matrix ($h_{a}^{2}$ = 0.34; $h_{g}^{2}$ = 0.28). In contrast, the estimation of genomic heritability of the NPCI index ($h_{g}^{2}$ = 0.46) had a higher value than that based on pedigree ($h_{a}^{2}$ = 0.40).

**Table 1:** The most heritable spectral reflectance indices (SRIs), according to genomic-based heritability $\left(h_{g}^{2}\geq 0.5\right)$ measured in adult leaves of cyanogenic *Eucalyptus cladocalyx*.

SRIs	Formula	hg2(s.e.)
Leaf Rust Disease Severity Index 1 (LRDSI1)	6.9 × (R_605_/R_455_)–1.2	0.58(0.03)
Anthocyanin Reflectance Index (ARI)	(1/R_550_)–(1/R_700_)	0.54(0.04)
Browning Reflectance Index (BRI)	R_450_/R_690_	0.54(0.03)
Simple Ratio 7 (SR7)	R_440_/_R690_	0.53(0.02)
Blue Green Pigment Index (BGI)	R_450_/R_550_	0.52(0.02)
Edge green first derivative normalized difference (EGFN)	(max(D_650:750_)–max(D_500:550_))/ (max(D_650:750_) + max(D_500:550_))	0.50(0.01)
Edge green first derivative ratio (EGFNR)	max(D_650:750_)/max(D_500:550_)	0.50(0.01)
Gitelson and Merzlyak Index 1 (GMI1)	R_750_/R_550_	0.50(0.03)
Simple Ratio 3 (SR3)	R_750_/R_550_	0.50(0.02)

*Genomic- and pedigree-based heritability estimates for all SRIs are presented in Supplementary Table S1. s.e., standard error*.

**Table 2:** Estimates of pedigree-based heritability ($h_{a}^{2}$) and genomic heritability ($h_{g}^{2}$) of selected reflectance indices (SRIs): simple ratio 10 (SR10), normalized difference lignin index (NDLI), normalized difference nitrogen index (NDNI), normalized pigment chlorophyll index (NPCI), and hydrogen cyanide (HCN) content.

SRIs/Trait	Formula	ha2(s.e.)	ha2(s.e.)
SR10	R_685_/R_655_	0.41(0.002)	0.41(0.02)
NDLI	(log(1/ R_1,754_)−log(1/R_1,680_)/(log(1/R_1,754_) + log(1/R_1680_)	0.34(0.001)	0.28(0.001)
NDNI	(log(1/R_1,510_)−log(1/R_1,680_))/(log(1/R_1,510_) + log(1/R_1,680_))	0.28(0.001)	0.29(0.001)
NPCI	(R_680_−R_430_)/(R_680_ + R_430_)	0.40(0.002)	0.46(0.002)

*The letter R_n_ indicates the reflectance value at the nth wavelength used to calculate each of the SRIs. s.e., standard error; SRIs, Selected spectral reflectance indices: SR10, simple ratio 10, NDLI, Normalized difference lignin index, NDNI, Normalized difference nitrogen index, and NPCI, Normalized pigment chlorophyll ratio index*.

### Prediction of HCN Content in Leaves of Adult *Eucalyptus* Trees Using an Integrated Approach

The goodness-of-fit measures for all the prediction models of HCN content that consider spectral reflectance data, based on different Bayesian regression methods (i.e., Bayes B, Bayes C, and Bayesian ridge regression), are shown in Table 3. On average, the Bayes B method had the best goodness-of-fit statistics (deviance information criterion; DIC) for the majority of the study models. Therefore, based on this Bayesian method, predictive ability (PA) estimates ranged from 0.41 to 0.60 (Table 4). Models that included spectral reflectance data as regressors outperformed conventional genomic prediction models in terms of PA and goodness-of-fit measures (Table 4). In fact, the goodness of fit of the prediction model based exclusively on genomic data (Model 3) was significantly increased with the incorporation of spectral data (Models 8 and 10; ∆DIC = 30 and 70, respectively).

**Table 3:** Goodness-of-fit testing for all prediction models of HCN content that consider spectral reflectance data, based on different Bayesian regression methods: Bayes B, Bayes C, and Bayesian ridge regression (BRR).

Model	Method	SRI	DIC	PV_G_	PV_SR_	PV_A_
$y^{*}=Qv+Z_{3}h+\varepsilon$ (7)	Bayes B	–	−367.5	–	24.4	–
	Bayes C	–	−365.4	–	24.6	–
	BRR	–	−364.7	–	22.9	–
$y^{*}=Qv+Z_{2}g_{2}+Z_{3}h+\varepsilon$ (8)	Bayes B	–	−377.3	30.8	19.1	–
	Bayes C	–	−379.8	30.8	20.4	–
	BRR	–	−380.4	30.6	18.9	–
$y^{*}=Qv+Za+Z_{3}h+\varepsilon$ (9)	Bayes B	–	−389.1	–	27.0	20.1
	Bayes C	–	−389.5	-	28.1	20.3
	BRR	–	−390.1	–	26.8	20.9
$y^{*}=Qv+X_{i}\beta_{i}+Z_{3}h+\varepsilon$ (11)	Bayes B	All	−365.5	–	27.8	–
	Bayes C	All	−364.7	–	28.4	–
	BRR	All	−363.9	–	25.7	–
$y^{*}=Qv+Za+Z_{2}g_{2}+Z_{3}h+\varepsilon$ (13)	Bayes B	–	−395.2	21.9	24.1	11.5
	Bayes C	–	−392.5	22.1	24.9	11.3
	BRR	–	−388.1	21.6	22.4	12.1
$y^{*}=Qv+X_{i}\beta_{i}+Za+Z_{2}g_{2}+Z_{3}h+\varepsilon$ (14)	Bayes B	All	−392.7	22.2	22.9	12.4
	Bayes C	All	−390.8	22.4	24.1	11.2
	BRR	All	−391.1	22.2	22.3	12.2

*The goodness of fit was tested using the deviance information criterion (DIC). PV_G_, PV_SR_, and PV_A_ are the percentages of variation of HCN explained by the genomic, spectral reflectance information, and pedigree, respectively. SRIs, Selected spectral reflectance indices; SR10, simple ratio 10, NDLI, normalized difference lignin index, NDNI, Normalized difference nitrogen index, and NPCI, Normalized pigment chlorophyll ratio index*.

**Table 4:** Predictive ability (PA) and goodness-of-fit measures for all models used for predicting cyanide (HCN) content in *Eucalyptus* trees.

Model^*^	SRI	PA	DIC	PV_G_	PV_SR_	PV_A_
$y^{*}=Qv+Z_{2}g_{2}+\varepsilon$ (3)	–	0.47	−307.4	35.2	–	–
$y^{*}=P\rho +Za+\varepsilon$ (4)	–	0.41	−313.5	–	–	27.0
$y^{*}=Qv+Za+\varepsilon$(5)	–	0.41	−350.2			27.3
$y^{*}=Qv+Za+Z_{2}g_{2}+\varepsilon$(6)	–	0.47	−320.8	30.3	–	15.0
$y^{*}=Qv+Z_{3}h+\varepsilon$ (7)	–	0.59	−367.5	–	24.4	–
$y^{*}=Qv+Z_{2}g_{2}+Z_{3}h+\varepsilon$ (8)	–	0.58	−377.3	30.8	19.1	–
$y^{*}=Qv+Za+Z_{3}h+\varepsilon$ (9)	–	0.59	−389.1	–	27.0	20.1
$y^{*}=Qv+X_{i}\beta_{i}+Z_{2}g_{2}+\varepsilon$ (10)	All	0.56	−338.7	39.2	–	–
	SR10	0.57	−339.5	35.3	–	–
	NDLI	0.46	−306.8	34.1	–	–
	NDNI	0.48	−308.2	33.7	–	–
	NPCI	0.52	−324.0	34.5	–	–
$y^{*}=Qv+X_{i}\beta_{i}+Z_{3}h+\varepsilon$ (11)	All	0.60	−365.5	–	27.8	–
$y^{*}=Qv+X_{i}\beta_{i}+Za+Z_{2}g_{2}+\varepsilon$ (12)	All	0.57	−358.5	29.4	–	17.3
$y^{*}=Qv+Za+Z_{2}g_{2}+Z_{3}h+\varepsilon$ (13)	–	0.59	−395.2	21.9	24.1	11.5
$y^{*}=Qv+X_{i}\beta_{i}+Za+Z_{2}g_{2}+Z_{3}h+\varepsilon$ (14)	All	0.59	−392.7	22.2	22.9	12

*The goodness of fit was tested using the deviance information criterion (DIC). PV_G_, PV_SR_, and PV_A_ are the percentages of variation of HCN explained by the genomic, spectral reflectance information, and pedigree, respectively*.

* *Models based on the method Bayes B. SRIs: Selected spectral reflectance indices: simple ratio 10 (SR10), normalized difference lignin index (NDLI), normalized difference nitrogen index (NDNI), and normalized pigment chlorophyll ratio index (NPCI)*.

**Figure 2:**
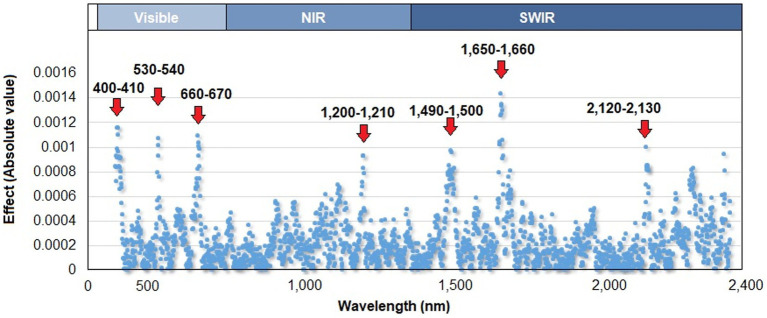
Absolute values of the wavelength effects along the reflectance curve (400–2,400 nm), estimated by the model with the best goodness-of-fit results (Model 13). The upper axis shows the three main regions of the spectrum: visible, near infrared (NIR), and shortwave infrared (SWIR).

The prediction model with the best goodness-of-fit measure (DIC = −395.2) was the one that included genomic, spectral reflectance, and pedigree information (Model 13), which had a predictive ability of 0.59. In terms of each component of Model 13, spectral reflectance data explained the greatest percentage of the phenotypic variation (24%), whereas genomic and pedigree data explained ~22% and ~ 12% of the total variation in HCN content, respectively. The model that included reflectance indices (SRIs; as covariates) showed a slight reduction in terms of goodness of fit (DIC = −392.7), whereas the PA value was maintained. The solution of wavelength effects accomplished using Model 13 is shown in Figure 2. Seven specific points of the reflectance spectrum had a greater effect on HCN: two regions within the visible spectrum (400–410 and 530–540 nm), one region within the visible–red edge spectral regions (660–670 nm) and four regions in the near and shortwave infrared regions (1,200–1,210, 1,490–1,500, 1,650–1,660, and 2,125–2,130.

Ten out of twelve models that included either spectral reflectance data or SRIs as regressors had a PA value above 0.5, whereas the models that exclusively included genomic and/or pedigree data had a PA below this value. The models that included the spectral reflectance component (*h*) had a PA value varying between 0.57 and 0.6. It should be noted that the goodness-of-fit statistics of Model 7 (exclusively based on spectral data) were increased by ~3 and 6% when genomic (Model 8) and pedigree (Model 9) data were included, respectively. The measures of the PA and goodness of fit of the model based exclusively on genomic data (Model 3; DIC = −307.4; PA = 0.47) were enhanced with the integration of genomic data and spectral reflectance indices (Model 10; DIC = −338.7; PA = 0.56). In particular, the prediction model that combined genomic data and the SR10 index had a lower DIC (DIC = −339.5) than the model based exclusively on genomic data and a 21% higher PA value. Additionally, the NPCI index increased the PA of model 3 by 10%, and improved its goodness of fit by 5% (DIC = −324.0). In contrast, the model that combined spectral data and SRIs (Model 11) had a lower goodness-of-fit statistic than the model based exclusively on spectral reflectance data (Model 7).

## Discussion

### Heritable Variation in Single Wavelengths

The spectral reflectance of the *E. cladocalyx* leaves showed a relatively moderate genetic control (*h*^2^ = 018–0.46) across the 400-to-2,400-nm spectral range. The heritability distribution along the reflectance curve indicates that additive gene effects fluctuate along the reflectance curve (Čepl et al., 2018). The reflectance regions with the highest genetic control (either genomic- or pedigree-based heritability) corresponded to the visible spectrum, between the wavelengths 460–490 and 575–590 nm, and the red edge region (700–715 nm). In agreement with this, Čepl et al. (2018), found that the maximum pedigree-based heritability value (i.e., *h*^2^ = 0.39) corresponded to the red edge inflection point (722 nm wavelength band) measured in a half-sib progeny test of *Pinus sylvestris*. The canopy reflectance at wavelengths between 400 and 1,000 nm is predominantly influenced by plant pigments (i.e., chlorophylls a and b) and cell structures (Schlerf et al., 2010; Raper and Varco, 2015; Zarco-Tejada et al., 2019). For instance, Schlerf et al. (2010), reported that the reflectance in the visible and red edge spectrum regions, measured in the canopy of *Picea abies*, can be used as a proxy for chlorophyll and nitrogen content. These findings can be observed in trees grown in different types of climates, such as tropical, Mediterranean, and oceanic climates (Schlerf et al., 2010; Li et al., 2019; Zarco-Tejada et al., 2019). In this context, chlorophyll content and other components in leaf cells could not only be regulated by abiotic and/or biotic factors, but also may be determined by inheritance patterns. Li et al. (2019) determined that the chlorophyll content and other variables related to the leaf color in *Sassafras tzumu* exhibit a high additive genetic control. In contrast, McKown et al. (2014) and Guerra et al. (2016) showed that ecophysiological parameters, such as chlorophyll content, photosynthetic rate, and nitrogen content, exhibit relatively low genetic control in *Populus* spp. This implies that the genetic control of the cellular components in the leaves (e.g., pigments) and their reflectance could be plant-specific. According to the results of this study, the cellular components of *E. cladocalyx* leaves, associated with reflectance between wavelengths 460 and 700 nm (e.g., chlorophylls and other pigments), could show relatively moderate genetic control.

In the present study, the estimates of the heritability of reflectance based on pedigree and genomic approaches in the short-wave infrared region (1,400–2,400 nm) were relatively lower than those in the visible spectrum region (including the red edge region). The reflectance in the short-wave infrared region has been associated with different primary and secondary metabolites, such as starch, celluloses, lignin, and other carbohydrates (Türker-Kaya and Huck, 2017; Čepl et al., 2019). In this sense, Paaso et al. (2017) reported that lignin exhibits a relatively low level of genetic control in senescent leaves of *Betula pendula*. Moreover, both the cellulose and lignin content in *Eucalyptus* wood are traits of moderate genetic control (Denis et al., 2013; Chen et al., 2018), which is consistent with the present study results.

### Heritable Variation in Spectral Reflectance Indices Associated With HCN Content in Leaves

Among the studied SRIs, only 3% of them were significantly associated with HCN. According to stepwise regression analysis, the SR10, NPCI, and NDLI indices were negatively correlated with HCN, whereas NDNI was positively correlated. These four selected SRIs had a similar pattern of heritability to those estimated at each wavelength. The indices calculated based on reflectance in the visible spectrum (i.e., SR10 and NPCI) showed greater heritable variation than the SRIs calculated within the SWNIR spectrum (i.e., NDLI and NDNI), which is in agreement with the heritability estimates in the different regions of the full spectrum (400–2,400 nm). SR10 (an index measured at 685 and 655 nm) has been previously related to chlorophylls and the efficient use of light (Chen et al., 2018). At the same time, the NPCI index was originally developed to evaluate the chlorophyll content in leaves (Peñuelas et al., 1994; Hatfield and Prueger, 2010). Previous studies have confirmed that this index could also be a proxy for the nitrogen content and could be an indicator of water stress and a good predictor of photochemical quenching (Filella et al., 1995; Huang et al., 2014; Maimaitiyiming et al., 2017). On the other hand, the NDLI index has been proposed as an indicator of lignin content (Serrano et al., 2002; Daughtry et al., 2004). However, it has also been considered to predict leaf biomass in rice (Cheng et al., 2017). According to Serrano et al. (2002), this index has been used as an indicator of the nitrogen content in leaves in different plants (Wang et al., 2016; Wang and Wei, 2016; Liang et al., 2018). In this context, most of the indices significantly correlated with HCN have been used to predict nitrogen content in leaves. According to Gleadow and Møller (2014), the content of cyanogenic glycosides is closely related to the availability of nitrogen in the soil, such that high doses of fertilizers increase the cyanogenic capacity of different crops. Relatedly, Gleadow and Woodrow (2000) and Simon et al. (2010) reported that the cyanogenic capacity of *E. cladocalyx* trees is increased with a greater availability of nitrogen in the soil (or substrate). Additionally, Mora-Poblete et al. (2021) reported a negative (although not significant) relationship between nitrogen and chlorophyll content (indirectly measured) and HCN in the leaves of *E. cladocalyx*. In fact, reflectance indices related to chlorophyll content can also be good predictors of nitrogen content in various tree species, such as *Picea*, *Acer*, and *Sorbus* (Wang et al., 2016).

Regarding the relationship between the NDLI index and HCN, the lignin content could be indirectly related to the HCN content. In fact, both prunasin and lignin correspond to secondary metabolites that come from a biosynthetic pathway that begins with the amino acid phenylalanine (Phe). The enzyme phenylalanine ammonia lyase converts Phe into cinnamate, which is subsequently converted to lignin through a series of enzymatic reactions. On the other hand, Phe can be hydroxylated by cytochrome P450 (in *E. cladocalyx*, CYP79A125; Hansen et al., 2018) and converted into phenylacetaldoxime, which is subsequently transformed into prunasin, suggesting a possible trade-off between the content of both secondary metabolites. On the other hand, these results should be interpreted with caution due to cyanogenic glycosides content competes for nitrogen with other nitrogen-containing compounds, since cyanogenic glycosides could account for up to 20% of leaf nitrogen (Hansen et al., 2018).

### Prediction of HCN Content in *Eucalyptus* Leaves

In this study, the most suitable model used to predict HCN in leaves of cyanogenic *Eucalyptus* was the one that included high-throughput phenotyping data, genomic information (haplotypes), and genealogical data (pedigree) in terms of the goodness-of-fit measurements. According to the results, the spectral reflectance explained a higher percentage of the HCN variation than both genomic and pedigree data, considering a half-sib progeny test. Consistently with these findings, Krause et al. (2019) reported that the prediction ability of a model based on a hyperspectral reflectance-derived relationship matrix could be equal to or greater than a prediction model that combined the genomic selection (GS) model with pedigree-derived relationship matrix (GS-A) for grain yields in wheat. Furthermore, the model that combined the three types of information was slightly superior to the GS-A model. In the present study, the prediction ability for HCN content based on spectral reflectance data (Model 7) was superior to that obtained by models based on either genomic or pedigree data. However, the model combining spectral reflectance, pedigree, and haplotype-based genomic data (Model 13) was not superior to Model 7 (in terms of its prediction ability). Gonçalves et al. (2021) reported that the prediction ability based on the spectral reflectance of the fiber and sucrose content of sugarcane stems was superior to that of the model based on genomic data alone. However, the combined use of spectral reflectance and genomic data did not significantly increase the prediction ability of the studied traits.

The hypothesis that a high-throughput phenotyping platform could indirectly capture endophenotypic variants can be related to specialized metabolites. Therefore, the expectation of a more robust prediction, considering the spectral reflectance relationship among trees, was confirmed. This result is consistent with that of Rincent et al. (2018), who showed how predicting the heading date in wheat based on spectral data is more accurate than that based on genomic information. In the same study, however, the prediction of wood-related traits in *Populus* trees based on spectral data was less precise than that based on genomic data. In another study, Sandhu et al. (2021) reported that the genomic prediction ability for protein content and grain yield in wheat could be increased by 12 and 20%, respectively, through the combined use of genomic data and SRIs. Interestingly, in the present study, the use of SRIs as covariates in predictive models of HCN led to an increase in the goodness-of-fit measures and the predictive ability of the GS model (Model 3). Therefore, the predictive capacities of both information resources could depend on the trait under study, the species, and the type of population. In practice, all types of information available must be tested. Indeed, in another study, Rutkoski et al. (2016) demonstrated that the use of spectral reflectance indices could increase GS precision by up to 70% for predicting the grain yield of wheat. It should be noted that none of the models that combined genomic data and SRIs was superior to the model based exclusively on spectral data in terms of prediction ability. However, SRIs may be relevant to explain the variation of HCN and the accuracy of the HCN prediction may require the use of various regions of the reflectance spectrum. In forage sorghum (*Sorghum bicolor*), Fox et al. (2012) determined that using a predictive model that combined the visible, NIR, and SW-NIR spectra allowed a better estimation of HCN than a model based on a single region of spectral signatures.

In general, the concentrations of different compounds in plants are estimated through the combined use of NIR spectral data with variable selection and dimensionality reduction models, such as PLS (Assis et al., 2017; Rizvi et al., 2018; Sampaio et al., 2018). However, the PLS method and other related ones are challenging to implement in genetically structured populations (genetic structures) or trials with complete pedigree information (Rincent et al., 2018; Krause et al., 2019). These types of information are relevant to the prediction process. In the present study case, three different Bayesian regression methods were evaluated, in which the Bayes B model offered a better goodness-of-fit measurement and a slightly higher prediction ability compared with both the BRR and Bayes C models. Bayes B is a regression method with a hierarchical Bayesian approach that performs the selection and reduction of the predictor variables (Meuwissen et al., 2001). Some studies have found that Bayesian regression models, such as Bayes B, have been more accurate than the PLS method (Solberg et al., 2009; Ferragina et al., 2015). Moreover, Gonçalves et al. (2021), reported that the Bayes B method was up to approximately two times more accurate than PLS in predicting fiber and sucrose con-tent in sugarcane stems. According to several studies (Kainer et al., 2018; Thistlethwaite et al., 2019; Rio et al., 2021), the Bayesian methods, such as Bayes B, can improve the predictive ability in genome -based evaluations. For instance, Kainer et al. (2018) evaluated the ability of different genomic prediction models of eight traits related to foliar terpene yield in *Eucalyptus polybractea*, using three different marker densities. According to these authors, Bayes B method outperformed ABLUP and GBLUP methods in the 54% of the total cases (total: 24 cases; 3 marker densities and 8 traits).

The Bayes B method allows for the identification of the most relevant variables to explain the variation of a response variable. In the present study, seven subregions of the spectral reflectance curve had a relatively greater effect on HCN (i.e., two, one, one, and five subregions of the reflectance curve: visible, red edge, near infrared, and shortwave infrared, respectively). Consistently, the SRIs significantly associated with HCN (i.e., SR10, NPCI, NDLI, and NDNI) were calculated from reflectance measurements located within (and near) these seven subregions of the spectrum. For example, the SR10 and NPCI indices were calculated from reflectance measurements located between the visible spectrum and in the red edge region (680 and 685 nm), whereas the NDNI and NDLI indices were calculated from reflectance measurements at 1680, 1510, and 430 nm. Based on the HCN prediction analysis, considering the model with the best good-ness-of-fit (Model 13), the spectral reflectance data explained the highest percentage of the phenotypic variation (24%), whereas genomic and pedigree components explained ~22 and 12% of the total variations in HCN content, respectively. These results suggest that the variation in the HCN content in *Eucalyptus* leaves could be mainly related to the spectral signature of the individual rather than its genotypic characterization based on haplotypes. However, several factors could affect the accuracy of genomic prediction models, including marker density (Lorenz and Smith, 2015). The density of markers (either haplotype and/or SNPs) used in the present study could be considered relatively low compared to other species of the genus *Eucalyptus* (Ballesta et al., 2020). In this sense, a greater number of markers should be included to address a greater number of regions in the genome explaining the variation of a specialized metabolite, mainly due to the quantitative nature of its genetic control (Mora-Poblete et al., 2021). Genomic prediction models have shown low to high prediction ability of secondary metabolites in trees (Kainer et al., 2018; Yamashita et al., 2020; Mora-Poblete et al., 2021). Our study proposes an alternative method to predict specialized metabolites in plants, which may be relevant in an economic, ecological and/or human health context. Yamashita et al. (2020) developed genomic prediction models for tea quality-related metabolites in 150 tea accessions, in which six models produced moderate prediction accuracy values for epigallocatechin gallate and caffeine, and low for free amino acids and chlorophylls. Kainer et al. (2018), compared several methods of genomic prediction for eight traits related to foliar terpene yield in *Eucalyptus polybractea*. PA was higher for the individual compounds, such as foliar α-pinene and 1,8-cineole concentration, than total foliar oil concentration, but the PA was significantly increased as the number of markers increased in most traits.

## Conclusion

This is the first study to examine heritable variation along the reflectance curve (in the range of 400–2,400 nm) and several spectral reflectance indices measured in leaves of a cyanogenic *Eucalyptus*. According to results, the variation in reflectance measurements and indices could be partially genetically driven, such that this information may help to recognize genotypes with specific chemical properties, such as cyanogenic capacity in leaves. The spectral reflectance of leaves of *E. cladocalyx* showed a relatively moderate level of genetic control. Consequently, we found four indices to be significantly associated with HCN content, all of which have been previously associated with nitrogen content in leaves, which agrees with previous studies examining the relationship between nitrogen and HCN in *Eucalyptus* leaves.

The model with the best fit to predict HCN content in leaves of cyanogenic *Eucalyptus* was the one that included spectral reflectance data, genomic information (haplotypes), and genealogical data (pedigree), as determined in terms of goodness-of-fit measures. In particular, its ability to predict HCN content in *Eucalyptus* leaves based exclusively on spectral data was superior to that obtained by models based on genomic and/or pedigree information. The strategy of prediction that integrates conventional pedigree information and genomic data, along with high-throughput phenotyping techniques, may be beneficial when the genome coverage is low or when the number of molecular markers is limited to predict a complex trait, such as secondary metabolites. Finally, we confirmed that the high-throughput phenotyping platforms indirectly capture endophenotypic variants related to secondary metabolites. Therefore, a more robust prediction can be made, considering the spectral reflectance in cyanogenic plant species.

## Data Availability Statement

The original contributions presented in the study are included in the article/Supplementary Material, further inquiries can be directed to the corresponding author.

## Author Contributions

FM-P contributed to conceptualization, done funding acquisition, and provided resources. PB and FM-P provided methodology, performed writing—original draft preparation, contributed to project administration, and performed investigation. PB provided software and carried out formal analysis. PB, SA, GL, and FJ-A performed validation. GL, SA, DM-C, and FJ-A performed data curation. FM-P, FJ-A, GL, and DM-C performed writing—review and editing. GL, FM-P, and FJ-A done supervision. All authors contributed to the article and approved the submitted version.

## Funding

This study was supported by FONDECYT (grant number 1201973).

## Conflict of Interest

The authors declare that the research was conducted in the absence of any commercial or financial relationships that could be construed as a potential conflict of interest.

## Publisher’s Note

All claims expressed in this article are solely those of the authors and do not necessarily represent those of their affiliated organizations, or those of the publisher, the editors and the reviewers. Any product that may be evaluated in this article, or claim that may be made by its manufacturer, is not guaranteed or endorsed by the publisher.
