# Supplementary material for: Heritable Variation of Foliar Spectral Reflectance Enhances Genomic Prediction of Hydrogen Cyanide in a Genetically Structured Population of Eucalyptus
Source: Front Plant Sci. 2022 Mar 31;13:871943. doi: 10.3389/fpls.2022.871943 (PMC9008590; doi:10.3389/fpls.2022.871943)
Supplement: Supplementary file 1 [file Table_1.DOCX]

Supplementary Material

**Supplementary Tables**

**Supplementary Table 1.** Estimates of genomic heritability ($h_{g}^{2}$) and pedigree-based heritability ($h_{a}^{2}$) of the Spectral Reflectance Indices (SRIs) calculated in adult leaves of cyanogenic eucalypt.

| **SRIs** | **Name** | **Formula** | $h_{g}^{2}$ | $h_{a}^{2}$ | **Reference** |
| --- | --- | --- | --- | --- | --- |
| ARI | Anthocyanin Reflectance Index | (1/R_550_) - (1/R_700_) | 0.54 | 0.34 | (Gitelson et al., 2001) |
| ARI2 | Anthocyanin Reflectance index 2 | R_800_(1/R_550_) - (1/R_700_) | 0.49 | 0.38 | (Gitelson et al., 2001) |
| BGI | Blue Green Pigment Index | R_450_/R_550_ | 0.52 | 0.35 | (Zarco-Tejada et al., 2005) |
| Boochs | Single Band 703 Boochs | D_703_ | 0.36 | 0.38 | (Boochs et al., 1990) |
| Boochs2 | Single Band 720 Boochs 2 | D_720_ | 0.37 | 0.43 | (Boochs et al., 1990) |
| BRI | Browning Reflectance Index | R_450_/R_690_ | 0.54 | 0.35 | (Zarco-Tejada et al., 2005) |
| CAI | Cellulose Absorption Index | 0.5 * (R_2000_ + R_2200_) – R_2100_ | 0.29 | 0.30 | (Nagler et al., 2003) |
| CARI | Chlorophyll Absorption Ratio Index | R_700_ * abs(a * 670 + R_670_ + b)/R_670_ * (a^2^ + 1)^0.5^ | 0.37 | 0.44 | (McMurtrey Iii et al., 1994) |
| Carter | Carter | R_695_/R_420_ | 0.46 | 0.36 | (Carter, 1994) |
| Carter2 | Carter 2 | R_695_/R_760_ | 0.39 | 0.43 | (Carter, 1994) |
| Carter3 | Carter 3 | R_605_/R_760_ | 0.41 | 0.44 | (Carter, 1994) |
| Carter4 | Carter 4 | R_710_/R_760_ | 0.38 | 0.45 | (Carter, 1994) |
| Carter5 | Carter 5 | R_695_/R_670_ | 0.39 | 0.28 | (Carter, 1994) |
| Carter6 | Carter 6 | R_550_ | 0.43 | 0.40 | (Carter, 1994) |
| CI | Coloration Index | R_675 *_ R_690_/R^2^_683_ | 0.32 | 0.33 | (Zarco-Tejada et al., 2003) |
| CI2 | Coloration Index 2 | R_760_/R_700_ - 1 | 0.38 | 0.44 | (Gitelson et al., 2003) |
| ClAInt | - |  | 0.40 | 0.44 | (Oppelt and Mauser, 2004) |
| CRI 1 | Carotenoid Reflectance Index 1 | 1/R_515_ - 1/R_550_ | 0.44 | 0.38 | (Gitelson et al., 2003) |
| CRI 2 | Carotenoid Reflectance Index 2 | 1/R_515_ - 1/R_770_ | 0.49 | 0.37 | (Gitelson et al., 2003) |
| CRI 3 | Carotenoid Reflectance Index 3 | 1/R_515_ - 1/R_550 *_ R_770_ | 0.43 | 0.44 | (Gitelson et al., 2003) |
| CRI 4 | Carotenoid Reflectance Index 4 | 1/R_515_ - 1/R_700 *_ R_770_ | 0.44 | 0.41 | (Gitelson et al., 2003) |
| D1 | Derivative index 1 | D_730_/D_706_ | 0.32 | 0.44 | (Zarco-Tejada et al., 2003) |
| D2 | Derivative index 2 | D_705_/D_722_ | 0.37 | 0.47 | (Zarco-Tejada et al., 2003) |
| Datt1 | Datt | (R_850_ - R_710_)/(R_850_ - R_680_) | 0.39 | 0.47 | (Datt, 1999) |
| Datt2 | Datt 2 | R_850_/R_710_ | 0.39 | 0.46 | (Datt, 1999) |
| Datt3 | Datt 3 | D_754_/D_704_ | 0.31 | 0.40 | (Datt, 1999) |
| Datt4 | Datt 4 | R_672_/(R_550 *_ R_708_) | 0.41 | 0.31 | (Datt, 1998) |
| Datt5 | Datt 5 | R_672_/R_550_ | 0.46 | 0.34 | (Datt, 1998) |
| Datt6 | Datt 6 | R_860_/(R_550 *_ R_708_) | 0.42 | 0.45 | (Datt, 1998) |
| Datt7 | Datt 7 | (R_860_ - R_2218_)/(R_860_ - R_1928_) | 0.26 | 0.23 | (Datt, 1998) |
| Datt8 | Datt 8 | (R_860_ - R_1788_)/(R_860_ - R_1928_) | 0.26 | 0.36 | (Datt, 1998) |
| DD | Double Difference Index | (R_749_ - R_720_) - (R_701_ - R_672_) | 0.40 | 0.47 | le Maire, François, and Dufrêne, 2004 |
| DDn | New Double Difference Index | 2 _*_ (R_710_ - R_660_ - R_760_) | 0.33 | 0.36 | (Le Maire et al., 2008) |
| DPI | Double Peak Index | (D_688_ - D_710_)/D^2^_697_ | 0.35 | 0.36 | (Zarco-Tejada et al., 2003) |
| DWSI1 | Disease water stress index 1 | R_800_/R_1660_ | 0.27 | 0.35 | (Apan et al., 2004) |
| DWSI2 | Disease water stress index 2 | R_1660_/R_550_ | 0.44 | 0.43 | (Apan et al., 2004) |
| DWSI3 | Disease water stress index 3 | R_1660_/R_680_ | 0.40 | 0.39 | (Apan et al., 2004) |
| DWSI4 | Disease water stress index 4 | R_550_/R_680_ | 0.43 | 0.32 | (Apan et al., 2004) |
| DWSI5 | Disease water stress index 5 | (R_800_ + R_550_)/(R_1660_ + R_680_) | 0.28 | 0.31 | (Apan et al., 2004) |
| EGFN | Edge green first derivative normalized difference | (max(D_650:750_) - max(D_500:550_))/ (max(D_650:750_) + max(D_500:550_)) | 0.50 | 0.35 | (Peñuelas et al., 1994) |
| EGFNR | Edge green first derivative ratio | max(D_650:750_)/ max(D_500:550_) | 0.50 | 0.35 | (Peñuelas et al., 1994) |
| EVI | Enhanced Vegetation Index | 2.5 _*_ ((R_800_ - R_670_)/(R_800_ - (6 _*_ R_670_) - (7.5 _*_ R_475_) + 1)) | 0.22 | 0.18 | (Huete et al., 1997) |
| GDVI_2 | Green Difference Vegetation Index 2 | (R^2^_800_ - R^2^_680_)/(R^2^_800_ + R^2^_680_) | 0.40 | 0.40 | (Wu, 2014) |
| GDVI_3 | Green Difference Vegetation Index 3 | (R^3^_800_ - R^3^_680_)/(R^3^_800_ + R^3^_680_) | 0.41 | 0.41 | (Wu, 2014) |
| GDVI_4 | Green Difference Vegetation Index 4 | (R^4^_800_ - R^4^_680_)/(R^4^_800_ + R^4^_680_) | 0.42 | 0.42 | (Wu, 2014) |
| GI | Greenness Index | R_554_/R_677_ | 0.43 | 0.32 | (Smith et al., 1995) |
| Gitelson | Gitelson | 1/R_700_ | 0.38 | 0.45 | (Gitelson et al., 1999) |
| Gitelson 2 | Gitelson 2 | (R_750_ - R_800_)/(R_695_ - R_740_) - 1 | 0.39 | 0.43 | (Gitelson et al., 2003) |
| GMI1 | Gitelson and Merzlyak Index 1 | R_750_/R_550_ | 0.50 | 0.41 | (Gitelson and Merzlyak, 1998) |
| GMI2 | Gitelson and Merzlyak Index 2 | R_750_/R_700_ | 0.38 | 0.44 | (Gitelson and Merzlyak, 1998) |
| Green_NDVI | Green Normalized Difference Vegetation Index | (R_800_ - R_550_)/(R_800_ + R_550_) | 0.49 | 0.42 | (Gitelson et al., 1996) |
| LRDSI1 | Leaf Rust Disease Severity Index 1 | 6.9*(R_605_/R_455_)-1.2 | 0.58 | 0.33 | (Ashourloo et al., 2014) |
| LWVI1 | Normalized Difference 1094/983 Leaf water VI 1 | (R_1094_ - R_983_) / (R_1094_ + R_983_) | 0.26 | 0.31 | (Galvao et al., 2005) |
| LWVI2 | Normalized Difference 1094/1205 Leaf water VI 2 | (R_1094_ - R_1205_) / (R_1094_ + R_1205_) | 0.22 | 0.31 | (Galvao et al., 2005) |
| Maccioni | Maccioni | (R_780_ - R_710_)/(R_780_ - R_680_) | 0.38 | 0.46 | (Maccioni et al., 2001) |
| MCARI | Modified Chlorophyll Absorption in Reflectance Index | ((R_700_ - R_670_) - 0.2 _*_ (R_700_ - R_550_)) _*_ (R_700_/R_670_) | 0.36 | 0.30 | (Daughtry et al., 2000) |
| MCARI/OSAVI | MCARI2/OSAVI2 | MCARI2/OSAVI | 0.39 | 0.42 | (Daughtry et al., 2000) |
| MCARI2 | Modified Chlorophyll Absorption in Reflectance Index 2 | ((R_750_ - R_705_) - 0.2 * (R_750_ -R_550_)) * (R_750_/R_705_) | 0.37 | 0.42 | (Haboudane et al., 2004) |
| MCARI2/OSAVI2 | MCARI2/OSAVI2 | MCARI2/OSAVI2 | 0.40 | 0.38 | (Wu et al., 2008) |
| mND705 | - | (R_750_-R_705_)/(R_750_ + R_705_-2* R_445_) | 0.38 | 0.44 | (Sims and Gamon, 2002) |
| mNDVI | Modified NDVI | (R_800_ - R_680_)/(R_800_ + R_680_ – 2 _*_ R_445_) | 0.39 | 0.38 | (Sims and Gamon, 2002) |
| MPRI | Modified Photochemical Reflectance Index | (R_515_ - R_530_)/(R_515_ + R_530_) | 0.44 | 0.40 | (Hernández-Clemente et al., 2011) |
| mREIP | Modified Red-Edge Inflection Point | mREIP with inverted Gaussian fit on reflectance | 0.40 | 0.45 | (Miller et al., 1990) |
| mSAVI | Modified Soil Adjusted Vegetation Index | 0.5 _*_ (2 _*_ R_800_ + 1- ((2 _*_ R_800_ + 1)^2^ – 8 _*_ (R_800_ - R_670_))^0.5^) | 0.38 | 0.38 | (Qi et al., 1994) |
| MSI | Moisture Stress Index | R_1600_/R_817_ | 0.26 | 0.35 | (Hunt Jr and Rock, 1989) |
| mSR | modified Simple Ratio | (R_800_−R_445_)/(R_680_−R_445_) | 0.39 | 0.36 | (Sims and Gamon, 2002) |
| mSR2 | modified Simple Ratio 2 | (R_750_/R_705_) -1/(R_750_/R_705_) + 1)^0.5^ | 0.38 | 0.43 | (Chen, 1996) |
| mSR705 | modified Simple Ratio 705 | (R_750_ - R_445_)/(R_705_-R_445_) | 0.38 | 0.44 | (Sims and Gamon, 2002) |
| MTCI | MERIS Terrestrial Chlorophyll Index | (R_754_ - R_709_)/(R_709_ - R_681_) | 0.37 | 0.45 | (Dash and Curran, 2004) |
| mTVI | modified Triangular Vegetation Index | 1.2 _*_ (1.2 _*_ (R_800_ - R_550_) - 2.5 _*_ (R_670_ - R_550_)) | 0.33 | 0.34 | (Haboudane et al., 2004) |
| NDLI | Normalized Difference Lignin Index | (log(1/ R_1754_) - log(1/ R_1680_))/(log(1/ R_1754_) + log(1/ R_1680_) | 0.28 | 0.34 | (Serrano et al., 2002) |
| NDNI | Normalized Difference Nitrogen Index | (log(1/ R_1510_) - log(1/ R_1680_))/(log(1/ R_1510_) + log(1/ R_1680_)) | 0.29 | 0.28 | (Serrano et al., 2002) |
| NDRE | Normalised difference red-edge | (R_730_ − R_780_)/(R_730_ + R_780_) | 0.37 | 0.46 | (Gitelson and Merzlyak, 1994; Sims and Gamon, 2002) |
| NDVI | Normalized Difference Vegetation Index | (R_800_ - R_680_)/(R_800_ + R_680_) | 0.38 | 0.39 | (Tucker, 1979) |
| NDVI2 | Normalized Difference Vegetation Index 2 | (R_750_ - R_705_)/(R_750_ + R_705_) | 0.38 | 0.44 | (Gitelson and Merzlyak, 1994) |
| NDVI3 | Normalized Difference Vegetation Index 3 | (R_682_ - R_553_)/(R_682_ + R_553_) | 0.45 | 0.33 | (Gandia et al., 2004) |
| NDWI | Normalized Difference Water Index | (R_857_-R_2130_)/(R_857_-R_2130_) | 0.25 | 0.39 | (Gao, 1996) |
| NPCI | Normalized Pigment Chlorophyll Index | (R_680_ - R_430_)/(R_680_ + R_430_) | 0.46 | 0.40 | (Peñuelas et al., 1994) |
| NPQI | Phaeophytinization Index | (R_415_-R_435_)/(R_415_+R_435_) | 0.35 | 0.35 | (Zarco-Tejada et al., 2001) |
| NRI | Nitrogen Reflectance Index | (R_570_ - R_670_)/(R_570_ + R_670_) | 0.44 | 0.32 | (Eaton et al., 2013) |
| OSAVI | Optimized Soil Adjusted Vegetation Index | (1 + 0.16) _*_ (R_800_-R_670_)/(R_800_ + R_670_ + 0.16) | 0.39 | 0.39 | (Rondeaux et al., 1996) |
| OSAVI2 | Optimized Soil Adjusted Vegetation Index 2 | (1 + 0.16) _*_ (R_750_-R_705_)/(R_750_ + R_705_ + 0.16) | 0.38 | 0.44 | (Wu et al., 2008) |
| RARS | Ratio Analysis of Reflectance Spectra | R_746_/R_513_ | 0.45 | 0.39 | (Chappelle et al., 1992) |
| PNC | Plant Nitrogen Concentation | R_700_/R_670_ | 0.39 | 0.29 | (McMurtrey Iii et al., 1994) |
| PRI | Photochemical Reflectance Index | (R_531_ - R_570_)/(R_531_ + R_570_) | 0.40 | 0.35 | (Gamon et al., 1992) |
| PRI*CI2 | PRI*CI2 | PRI*CI2 | 0.37 | 0.29 | (Garrity et al., 2011) |
| PRI_norm | normalized PRI | PRI _*_ (-1)/(RDVI _*_ R_700_/R_670_) | 0.41 | 0.39 | (Zarco-Tejada et al., 2013) |
| PSND | Pigment specific normalised difference | (R_800_ - R_470_)/(R_800_ + R_470_) | 0.48 | 0.42 | (Blackburn, 1998) |
| PSRI | Plant Senescence Reflectance Index | (R_678_ - R_500_)/R_750_ | 0.47 | 0.34 | (Merzlyak et al., 1999) |
| PSSR | Pigment specific simple ratio | R_800_/R_635_ | 0.39 | 0.41 | (Blackburn, 1998) |
| PWI | Plant Water Index | R_900_/R_970_ | 0.31 | 0.37 | (Peñuelas et al., 1997) |
| RDVI | Renormalized Difference Vegetation Index | (R_800_ - R_670_)/SQRT(R_800_ + R_670_) | 0.36 | 0.38 | (Roujean and Breon, 1995) |
| REP_LE | - | Red-edge position through linear extrapolation | 0.40 | 0.48 | (Cho and Skidmore, 2006) |
| REP_Li | - | 700 + 40 · ((R_re_ − R_700_)/(R_740_ − R_700_)) | 0.41 | 0.47 | (Guyot and Baret, 1988) |
| RGI | Red/Green Index | R_690_/R_550_ | 0.48 | 0.34 | (Zarco-Tejada et al., 2005) |
| SAVI | Soil Adjusted Vegetation Index | (1 + L) _*_ (R_800_ - R_670_)/(R_800_ + R_670_ + L) | 0.38 | 0.39 | (Huete, 1988) |
| SIPI | Structure Intensive Pigment Index | (R_800_ - R_445_)/(R_800_ - R_680_) | 0.38 | 0.39 | (Peñuelas et al., 1995) |
| SPVI | Spectral Polygon Vegetation Index | 0.4 _*_ (3.7 _*_ (R_800_ | 0.37 | 0.43 | (Vincini et al., 2006) |
| SR | Simple Ratio | R_800_/R_680_ | 0.38 | 0.38 | (Jordan, 1969) |
| SR1 | Simple Ratio 1 | R_750_/R_700_ | 0.39 | 0.43 | (Gitelson and Merzlyak, 1997) |
| SR10 | Simple Ratio 10 | R_685_/R_655_ | 0.41 | 0.41 | (Zarco-Tejada et al., 2003) |
| SR2 | Simple Ratio 2 | R_752_/R_690_ | 0.38 | 0.41 | (Gitelson and Merzlyak, 1997) |
| SR3 | Simple Ratio 3 | R_750_/ R_550_ | 0.50 | 0.40 | (Gitelson and Merzlyak, 1997) |
| SR4 | Simple Ratio 4 | R_700_/ R_670_ | 0.39 | 0.30 | (McMurtrey Iii et al., 1994) |
| SR5 | Simple Ratio 5 | R_675_/R_700_ | 0.40 | 0.30 | (Chappelle et al., 1992) |
| SR6 | Simple Ratio 6 | R_750_/R_710_ | 0.38 | 0.45 | (Zarco‐Tejada and Miller, 1999) |
| SR7 | Simple Ratio 7 | R_440_/_R690_ | 0.53 | 0.37 | (Lichtenthaler et al., 1996) |
| SR8 | Simple Ratio 8 | R_515_/R_550_ | 0.49 | 0.33 | (Hernández-Clemente et al., 2012) |
| SR9 | Simple Ratio 9 | R_690_/R_655_ | 0.48 | 0.36 | (Zarco-Tejada et al., 2003) |
| SRPI | Simple Ratio Pigment Index | R_430_/R_680_ | 0.48 | 0.39 | (Pen Uelas et al., 1995) |
| SRWI | Simple Ratio 850/1240 | R_850_/R_1240_ | 0.25 | 0.39 | (Zarco-Tejada et al., 2003) |
| Sum_Dr1 | - | $\sum_{i=626}^{795} {D1}_{i}$ | 0.26 | 0.37 | (Elvidge and Chen, 1995) |
| Sum_Dr2 | - | $\sum_{i=680}^{780} {D1}_{i}$ | 0.30 | 0.38 | (Filella and Peñuelas, 1994) |
| SWIRFI | Shortwave-infrared | ((R_2133_)^2^ / R_2225_)*(R_2209_)^3^ | 0.35 | 0.30 | (Levin et al., 2007) |
| SWIRLI | Shortwave-infrared | 3.87*(R_2210_ - R_2090_) - 27.51*(R_2280_ - R_2090_) - 0.2 | 0.37 | 0.28 | (Lobell et al., 2001) |
| SWIRSI | Shortwave-infrared | −41.59 · (R_2210_ − R_2090_)+  1.24 · (R_2280_ − R_2090_) + 0.64 | 0.45 | 0.37 | (Lobell et al., 2001) |
| SWIRVI | Shortwave-infrared | 37.72*(R_2210_ - R_2090_) + 26.27*(R_2280_ - R_2090_) + 0.57 | 0.44 | 0.31 | (Lobell et al., 2001) |
| TCARI | Transformed Chlorophyll Absorbtion Ratio | 3 _*_ ((R_700_ -R_670_) - 0.2 _*_ (R_700_ -R_550_) _*_ (R_700_/R_670_)) | 0.39 | 0.41 | (Haboudane et al., 2002) |
| TCARI/OSAVI | TCARI/OSAVI | TCARI/OSAVI | 0.42 | 0.46 | (Haboudane et al., 2002) |
| TCARI2 | Transformed Chlorophyll Absorbtion Ratio 2 | 3 _*_ ((R_750_ - R_705_) - 0.2 _*_ (R_750_ -R_550_) _*_ (R_750_/R_705_)_)_ | 0.40 | 0.41 | (Wu et al., 2008) |
| TCARI2/OSAVI2 | TCARI2/OSAVI2 | TCARI2/OSAVI2 | 0.45 | 0.34 | (Wu et al., 2008) |
| TGI | Triangular greenness index | -0.5 _*_ (190 _*_ (R_670_ - R_550_) – 120 _*_ (R_670_ -R_480_)) | 0.46 | 0.33 | (Hunt Jr et al., 2013) |
| TVI | Transformed Vegetation Index | 0.5 _*_ (120 _*_ (R_750_ - R_550_) – 200 _*_ (R_670_ - R_550_)) | 0.32 | 0.34 | (Broge and Leblanc, 2001) |
| Vogelmann | Vogelmann indices | R_740_/R_720_ | 0.37 | 0.45 | (Vogelmann et al., 1993) |
| Vogelmann2 | Vogelmann indices 2 | (R_734_ -R_747_)/(R_715_ + R_726_) | 0.36 | 0.45 | (Vogelmann et al., 1993) |
| Vogelmann3 | Vogelmann indices 3 | D_715_/D_705_ | 0.39 | 0.46 | (Vogelmann et al., 1993) |
| Vogelmann4 | Vogelmann indices 4 | (R_734_ - R_747_)/(R_715_ + R_720_) | 0.36 | 0.45 | (Vogelmann et al., 1993) |
| VS | Vegetation Stress ratio | R_725_/ R_702_ | 0.38 | 0.43 | (Smith et al., 2004) |
| WI | Water Index | R_900_/R_970_ | 0.31 | 0.37 | (Peñuelas et al., 1993) |

**References**

Apan, A., Held, A., Phinn, S., Markley, J. (2004). Detecting sugarcane ‘orange rust’disease using EO-1 Hyperion hyperspectral imagery. Int. J. Remote Sens. 25, 489–498.

Ashourloo, D., Mobasheri, M.R., Huete, A. (2014). Developing two spectral disease indices for detection of wheat leaf rust (Pucciniatriticina). Remote Sens. 6, 4723–4740.

Blackburn, G.A. (1998). Quantifying chlorophylls and caroteniods at leaf and canopy scales: An evaluation of some hyperspectral approaches. Remote Sens. Environ. 66, 273–285.

Boochs, F., Kupfer, G., Dockter, K., Kühbauch, W. (1990). Shape of the red edge as vitality indicator for plants. Remote Sens. 11, 1741–1753.

Broge, N.H., and Leblanc, E. (2001). Comparing prediction power and stability of broadband and hyperspectral vegetation indices for estimation of green leaf area index and canopy chlorophyll density. Remote Sens. Environ. 76, 156–172.

Carter, G.A. (1994). Ratios of leaf reflectances in narrow wavebands as indicators of plant stress. Remote Sens. 15, 697–703.

Chappelle, E.W., Kim, M.S., McMurtrey, III J.E. (1992). Ratio analysis of reflectance spectra (RARS): an algorithm for the remote estimation of the concentrations of chlorophyll a, chlorophyll b, and carotenoids in soybean leaves. Remote Sens. Environ. 39, 239–247.

Chen, J.M. (1996). Evaluation of vegetation indices and a modified simple ratio for boreal applications. Can. J. Remote Sens. 22, 229–242.

Cho, M.A., and Skidmore, A.K. (2006). A new technique for extracting the red edge position from hyperspectral data: The linear extrapolation method. Remote Sens. Environ. 101, 181–193.

Dash, J., and Curran, P.J. (2004). The MERIS terrestrial chlorophyll index. Int. J. Remote Sens. 25, 5403-5413.

Datt, B. (1998). Remote sensing of chlorophyll a, chlorophyll b, chlorophyll a+ b, and total carotenoid content in eucalyptus leaves. Remote Sens. Environ. 66, 111–121.

Datt, B. (1999). Remote sensing of water content in Eucalyptus leaves. Aust. J. Botany 47, 909–923.

Daughtry, C.S.T., Walthall, C.L., Kim, M.S., De Colstoun, E.B., McMurtreyIii, J.E. (2000). Estimating corn leaf chlorophyll concentration from leaf and canopy reflectance. Remote Sens. Environ. 74, 229–239.

Eaton, S.L., Roche, S.L., Llavero-Hurtado, M., Oldknow, K.J., Farquharson, C., Gillingwater, T.H., et al. (2013). Total protein analysis as a reliable loading control for quantitative fluorescent Western blotting. PloS One 8, e72457.

Elvidge, C.D., and Chen, Z. (1995). Comparison of broad-band and narrow-band red and near-infrared vegetation indices. Remote Sens. Environ. 54, 38–48.

Filella, I., and Peñuelas J. (1994). The red edge position and shape as indicators of plant chlorophyll content, biomass and hydric status. Int. J. Remote Sens. 15, 1459–1470.

Galvao, L.S., Formaggio, A.R., Tisot, D.A. (2005). Discrimination of sugarcane varieties in Southeastern Brazil with EO-1 Hyperion data. Remote Sens. Environ. 94, 523–534.

Gamon, J.A., Peñuelas, J., Field, C.B. (1992). A narrow-waveband spectral index that tracks diurnal changes in photosynthetic efficiency. Remote Sens. Environ. 41, 35–44.

Gandia, S., Fernández, G., García, J.C., Moreno, J. (2004). Retrieval of vegetation biophysical variables from CHRIS/PROBA data in the SPARC campaign. Esa. Sp. 578, 40–48.

Gao, B-C. (1996). NDWI—A normalized difference water index for remote sensing of vegetation liquid water from space. Remote Sens. Environ. 58, 257–266.

Garrity, S.R., Eitel, J.U.H., Vierling, L.A. (2011). Disentangling the relationships between plant pigments and the photochemical reflectance index reveals a new approach for remote estimation of carotenoid content. Remote Sens. Environ. 115, 628–635.

Gitelson, A.A., Buschmann, C., Lichtenthaler, H.K. (1999). The chlorophyll fluorescence ratio F735/F700 as an accurate measure of the chlorophyll content in plants. Remote Sens. Environment 69, 296–302.

Gitelson, A.A., Gritz, Y., Merzlyak, M.N. (2003). Relationships between leaf chlorophyll content and spectral reflectance and algorithms for non-destructive chlorophyll assessment in higher plant leaves. J. Plant Physiol. 160, 271–282.

Gitelson, A.A., Kaufman, Y.J., Merzlyak, M.N. (1996). Use of a green channel in remote sensing of global vegetation from EOS-MODIS. Remote Sens. Environ. 58, 289–298.

Gitelson, A., and Merzlyak, M.N. (1994). Quantitative estimation of chlorophyll-a using reflectance spectra: Experiments with autumn chestnut and maple leaves. J. Photochem. Photobiol. B: Biology 22, 247–252.

Gitelson, A.A., and Merzlyak, M.N. (1997). Remote estimation of chlorophyll content in higher plant leaves. Int. J. Remote Sens. 18, 2691–2697.

Gitelson, A.A., and Merzlyak, M.N. (1998). Remote sensing of chlorophyll concentration in higher plant leaves. Adv. Space Res. 22, 689–692.

Gitelson, A.A., Merzlyak, M.N., Chivkunova, O.B. (2001). Optical properties and nondestructive estimation of anthocyanin content in plant leaves¶. Photochem. Photobiology 74, 38–45.

Guyot, G., and Baret, F. (1988). Utilization de la haute resolution spectrale pour suivre l’etat des couverts vegetaux. Spec. Sign. Obj. Remote Sens. 287, 279.

Haboudane, D., Miller, J.R., Tremblay, N., Pattey, E., Vigneault, P. (2004). Estimation of leaf area index using ground spectral measurements over agriculture crops: Prediction capability assessment of optical indices. XXth ISPRS Congress:" Geo-Imagery Bridging Continents". Istanbul, Turkey.12–23.

Haboudane, D., Miller, J.R., Tremblay, N., Zarco-Tejada, P.J., Dextraze, L. (2002). Integrated narrow-band vegetation indices for prediction of crop chlorophyll content for application to precision agriculture. Remote Sens. Environ. 81, 416–426.

Hernández-Clemente, R., Navarro-Cerrillo, R.M., Suárez, L., Morales, F., Zarco-Tejada, P.J. (2011). Assessing structural effects on PRI for stress detection in conifer forests. Remote Sens. Environ. 115, 2360–2375.

Hernández-Clemente, R., Navarro-Cerrillo, R.M., Zarco-Tejada, P.J. (2012). Carotenoid content estimation in a heterogeneous conifer forest using narrow-band indices and PROSPECT+ DART simulations. Remote Sens. Environ. 127, 298–315.

Huete, A.R. (1988). A soil-adjusted vegetation index (SAVI). Remote Sens. Environ. 25, 295-309.

Huete, A.R., Liu, H.Q., Batchily, K.V., Van Leeuwen, W. (1997). A comparison of vegetation indices over a global set of TM images for EOS-MODIS. Remote Sens. Environ. 59, 440–451.

Hunt Jr, E.R., Doraiswamy, P.C., McMurtrey, J.E., Daughtry, C.S.T., Perry, E.M., Akhmedov, B. (2013). A visible band index for remote sensing leaf chlorophyll content at the canopy scale. Int. J. Appl. Earth Observation Geoinfor. 21, 103–112.

Hunt Jr, E.R., and Rock, B.N. (1989). Detection of changes in leaf water content using near-and middle-infrared reflectances. Remote Sens. Environ. 30, 43–54.

Jordan, C.F. (1969). Derivation of leaf‐area index from quality of light on the forest floor. Ecology 50, 663–666.

Levin, N., Kidron, G.J., Ben‐Dor, E. (2007). Surface properties of stabilizing coastal dunes: combining spectral and field analyses. Sedimentology 54, 771–788.

Lichtenthaler, H.K., Lang, M., Sowinska, M., Heisel, F., Miehe, J.A. (1996). Detection of vegetation stress via a new high resolution fluorescence imaging system. J. Plant Physiol. 148, 599–612.

Lobell, D.B., Asner, G.P., Law, B.E., Treuhaft, R.N. (2001). Subpixel canopy cover estimation of coniferous forests in Oregon using SWIR imaging spectrometry. J. Geoph. Res.: Atmospheres 106, 5151–5160.

Maccioni, A., Agati, G., Mazzinghi, P. (2001). New vegetation indices for remote measurement of chlorophylls based on leaf directional reflectance spectra. J. Photochem. Photobiol. B: Biology 61, 52–61.

Le Maire, G., François, C., Soudani, K., Berveiller, D., Pontailler, J-Y., et al. (2008). Calibration and validation of hyperspectral indices for the estimation of broadleaved forest leaf chlorophyll content, leaf mass per area, leaf area index and leaf canopy biomass. Remote Sens. Environ. 112, 3846–3864.

McMurtreIii, J.E., Chappelle, E.W., Kim, M.S., Meisinger, J.J., Corp, L.A. (1994). Distinguishing nitrogen fertilization levels in field corn (Zea mays L.) with actively induced fluorescence and passive reflectance measurements. Remote Sens. Environ. 47, 36–44.

Merzlyak, M.N., Gitelson, A.A., Chivkunova, O.B., Rakitin, V.Y. (1999). Non‐destructive optical detection of pigment changes during leaf senescence and fruit ripening. Physiol. Plantarum 106, 135–141.

Miller, J.R., Hare, E.W., Wu, J. (1990). Quantitative characterization of the vegetation red edge reflectance 1. An inverted-Gaussian reflectance model. Remote Sens. 11, 1755–1773.

Nagler, P.L., Inoue, Y., Glenn, E.P., Russ, A.L., Daughtry, C.S.T. (2003). Cellulose absorption index (CAI) to quantify mixed soil–plant litter scenes. Remote Sens. Environ. 87, 310–325.

Oppelt, N., and Mauser, W. (2004). Hyperspectral monitoring of physiological parameters of wheat during a vegetation period using AVIS data. Int. J. Remote Sens. 25, 145–159.

Peñuelas, J., Filella, I., Lloret, P., Muñoz, F., Vilajeliu, M. (1995). Reflectance assessment of mite effects on apple trees. Int. J. Remote Sens. 16, 2727–2733.

Peñuelas, J., Baret, F., Filella, I. (1995). Semi-empirical indices to assess carotenoids/chlorophyll a ratio from leaf spectral reflectance. Photosynthetica 31, 221–230.

Peñuelas, J., Filella, I., Biel, C., Serrano, L., Save, R. (1993). The reflectance at the 950–970 nm region as an indicator of plant water status. Int. J. Remote Sens. 14, 1887–1905.

Peñuelas, J., Gamon, J.A., Fredeen, A.L., Merino, J., Field, C.B. (1994). Reflectance indices associated with physiological changes in nitrogen-and water-limited sunflower leaves. Remote Sens. Environ. 48, 135–146.

Peñuelas, J., Pinol, J., Ogaya, R., Filella, I. (1997). Estimation of plant water concentration by the reflectance water index WI (R900/R970). Int. J. Remote Sens. 18, 2869–2875.

Qi, J., Chehbouni, A., Huete, A.R., Kerr, Y.H., Sorooshian, S. (1994). A modified soil adjusted vegetation index. Remote Sens. Environ. 48, 119–126.

Rondeaux, G., Steven, M., Baret, F. (1996). Optimization of soil-adjusted vegetation indices. Remote Sens. Environ. 55, 95–107.

Roujean, J-L., and Breon, F-M. (1995). Estimating PAR absorbed by vegetation from bidirectional reflectance measurements. Remote Sens. Environ. 51, 375–384.

Serrano, L., Peñuelas, J., Ustin, S.L. (2002). Remote sensing of nitrogen and lignin in Mediterranean vegetation from AVIRIS data: Decomposing biochemical from structural signals. Remote Sens. Environ. 81, 355–364.

Sims, D.A., and Gamon, J.A. (2002). Relationships between leaf pigment content and spectral reflectance across a wide range of species, leaf structures and developmental stages. Remote Sens. Environ. 81, 337–354.

Smith, R.C.G., Adams, J., Stephens, D.J., Hick, P.T. (1995). Forecasting wheat yield in a Mediterranean-type environment from the NOAA satellite. Aust. J. Agr. Res. 46, 113–125.

Smith, K.L., Steven, M.D., Colls, J.J. (2004). Use of hyperspectral derivative ratios in the red-edge region to identify plant stress responses to gas leaks. Remote Sens. Environ. 92, 207–217.

Tucker, C.J. (1979). Red and photographic infrared linear combinations for monitoring vegetation. Remote Sens. Environ. 8, 127–150.

Vincini, M., Frazzi, E., D’Alessio, P. (2006). Angular dependence of maize and sugar beet VIs from directional CHRIS/Proba data. Proc. 4th ESA Chris Proba Workshop.19–21.

Vogelmann, J.E., Rock, B.N., Moss, D.M. (1993). Red edge spectral measurements from sugar maple leaves. Int. J. Remote Sens. 14, 1563–1575.

Wu, W. (2014). The generalized difference vegetation index (GDVI) for dryland characterization. Remote Sens. 6, 1211–1233.

Wu, C., Niu, Z., Tang, Q., Huang, W. (2008). Estimating chlorophyll content from hyperspectral vegetation indices: Modeling and validation. Agr. Forest Meteorol. 148, 1230–1241.

Zarco-Tejada, P.J., Berjón, A., López-Lozano, R., Miller, J.R., Martín, P., Cachorro, V., et al. (2005). Assessing vineyard condition with hyperspectral indices: Leaf and canopy reflectance simulation in a row-structured discontinuous canopy. Remote Sens. Environ. 99, 271–287.

Zarco-Tejada, P.J., González-Dugo, V., Williams, L.E., Suárez, L., Berni, J.A.J., Goldhamer, D., et al. (2013). A PRI-based water stress index combining structural and chlorophyll effects: Assessment using diurnal narrow-band airborne imagery and the CWSI thermal index. Remote Sens. Environ. 138, 38–50.

Zarco-Tejada, P.J., Miller, J.R., Noland, T.L., Mohammed, G.H., Sampson, P.H. (2001). Scaling-up and model inversion methods with narrowband optical indices for chlorophyll content estimation in closed forest canopies with hyperspectral data. IEEE T. Geosci. Remote 39, 1491–1507.

Zarco-Tejada, P.J., Pushnik, J.C., Dobrowski, S., Ustin, S.L. (2003). Steady-state chlorophyll a fluorescence detection from canopy derivative reflectance and double-peak red-edge effects. Remote Sens. Environ. 84, 283–294.

Zarco‐Tejada, P.J., and Miller, J.R. (1999). Land cover mapping at BOREAS using red edge spectral parameters from CASI imagery. J. Geophys. Res.: Atmospheres 104, 27921–27933.
